# Self-esteem and inner strengths: a network study in Thai university students with borderline personality disorder symptoms

**DOI:** 10.3389/fpsyt.2026.1847294

**Published:** 2026-07-07

**Authors:** Yuting Song, Justin DeMaranville, Kanyarat Khattiya, Kelvin Leung, Nahathai Wongpakaran, Tinakon Wongpakaran

**Affiliations:** 1 Mental Health Program, Multidisciplinary and Interdisciplinary School (MidS), Chiang Mai University, Chiang Mai, Thailand; 2 Mental Health and Psychiatry Services, Maesai Hospital, Chiang Rai, Thailand; 3 Faculty of Medicine and Health, The University of Sydney, Sydney, NSW, Australia; 4 Department of Psychiatry, Faculty of Medicine, Chiang Mai University, Chiang Mai, Thailand

**Keywords:** borderline personality features, cultural psychology, emerging adulthood, inner strengths, network analysis, protective factors, self-esteem, Ten Pāramīs

## Abstract

**Introduction:**

Self-esteem is widely regarded as an important construct in the psychological functioning of individuals with borderline personality disorder (BPD) symptoms. During emerging adulthood, fluctuations in self-esteem are often linked to emotional dysregulation and maladaptive adjustment; however, self-esteem has rarely been examined within a broader system of culturally relevant psychological resources. Drawing on the Theravāda Buddhist framework of the Ten Pāramīs, inner strengths may represent protective resources that can be examined alongside self-esteem to clarify how these strengths co-occur among students experiencing BPD symptoms.

**Methods:**

The present study employed a regularized psychological network approach to investigate partial associations between self-esteem and Ten Pāramī–based inner strengths among Thai university students screening positive for BPD symptoms. Participants were 346 Thai university students (25.4% male, 74.6% female; mean age = 21.60 ± 2.24 years) identified using a standardized BPD screening instrument. Inner strengths included Truthfulness, Perseverance, Wisdom, Generosity, adherence to the Five Precepts, Meditation, Tolerance, Equanimity, Determination, and Loving-kindness.

**Result:**

The estimated network showed a predominantly positive pattern of partial associations among strengths. The strongest edge was observed between Generosity and Loving-kindness, and links between self-esteem and Determination (and Equanimity) were among the most consistently estimated associations (based on bootstrap confidence intervals). Centrality indices were examined descriptively; however, case-dropping bootstrap results indicated very limited stability of centrality estimates (CS(cor = 0.7) = 0.13 for strength and expected influence), and centrality rankings were therefore treated as strictly exploratory. Discussion: Although some negative partial associations were estimated (e.g., involving Truthfulness, Perseverance, and Equanimity), their precision was limited and such patterns should be treated as exploratory. Exploratory gender-stratified analyses suggested that the strongest edges were similar in the female subsample, whereas the male subsample yielded a sparse/near-empty regularized network, limiting inference regarding gender differences. Given the cross-sectional design, all associations are interpreted as conditional co-occurrence rather than directional or causal effects.

**Conclusion:**

Overall, these findings highlight a small set of robust co-occurring inner strengths linked to self-esteem in Thai university students with BPD symptoms and provide a culturally informed basis for hypothesis generation regarding strengths-based skills cultivation and supportive interventions in university settings.

## 1 Introduction

Self-esteem, defined as an individual’s global evaluation of self-worth, occupies a central position in the psychopathology of borderline personality disorder (BPD) (1–4). Individuals with borderline personality features exhibit pronounced fluctuations in self-esteem (5, 6). Such instability not only undermines emotion regulation capacity but also amplifies the impact of external stressors and interpersonal conflicts, thereby contributing to a vicious cycle of psychological dysfunction (7). Previous research has demonstrated that both low levels and high variability of self-esteem are significantly associated with BPD symptoms and can predict clinical manifestations such as self-injurious behaviors, impulsivity, and affective outbursts (8, 9). Importantly, instability in self-esteem has been shown to discriminate individuals with BPD from other clinical populations more effectively than affective instability (2), suggesting that disturbances in self-esteem are not merely epiphenomena of emotional symptoms, but rather appear to represent a core component of BPD-related psychopathology and may be closely intertwined with broader dysregulation processes. This issue is particularly salient among university students. In Thailand, the prevalence of BPD symptoms among university students has been reported at 6.4% (10), markedly higher than that observed in the general population (11). University students are in the developmental stage of emerging adulthood, during which key developmental tasks include the establishment of a stable sense of identity and the formation of mature intimate relationships (12, 13). For individuals with borderline personality features, these tasks themselves constitute substantial psychological challenges. Empirical evidence indicates that university students with borderline personality features are more likely to experience affective instability, difficulties in impulse control, an increased risk of substance misuse, and engagement in self-injurious behaviors (13–16). These vulnerabilities not only exacerbate fluctuations in self-esteem but also increase susceptibility to depression and anxiety (17–19). Prior Thai student research further underscores the public health relevance of BPD symptom screening in university settings and the need to identify culturally congruent protective resources that may co-occur with self-worth in this population.

In this background, the concept of positive psychological resources has gained increasing attention. In recent years, researchers have begun to focus on protective factors that can buffer emotional symptoms and stabilize self-worth (20–22). Among these, the framework of inner strengths derived from the Theravāda Buddhist concept of the Ten Pāramīs has attracted particular interest. This framework encompasses ten psychological qualities: Truthfulness, Perseverance, Wisdom, Generosity, adherence to the Five Precepts, Meditation, Tolerance, Equanimity, Determination, and Loving-kindness (23). In regions where Theravāda Buddhism is culturally prominent, such as Thailand, this framework not only has deep cultural roots but also offers a culturally grounded perspective for understanding mental health (23–25). Conceptually, several Pāramī dimensions map onto self-worth regulation processes that are especially relevant to emerging adulthood and BPD vulnerability, including prosocial orientation (e.g., generosity/loving-kindness), values-consistent commitment and persistence (determination), and nonreactivity/balance under emotional volatility (equanimity).

As protective psychological resources, inner strengths have been shown to buffer emotional dysregulation, reduce rumination and self-injurious behaviors, and mitigate the adverse effects of neuroticism and perceived stress on mental health (24, 26–29). Previous studies have also provided preliminary evidence of a stable positive association between meditation and self-esteem (30), and individuals with higher levels of adherence to moral precepts have reported higher self-esteem (25). Collectively, these findings suggest that different dimensions of inner strengths may play important roles in stabilizing self-esteem.

However, the functional differentiation among the dimensions of inner strengths may be substantial, whereas existing studies have largely treated inner strengths as a homogeneous construct or have examined only single dimensions in isolation (4). Such approaches obscure this complexity and make it difficult to elucidate the specific patterns of association between self-esteem and the various dimensions of inner strengths (31).

Network analysis offers a powerful approach to uncover such systemic patterns of association. By conceptualizing psychological variables as interconnected nodes and representing their conditional dependencies as edges, network analysis enables the identification of central nodes and structural features within the system, as well as key variables that occupy central or bridging positions (32–34). In the present study, network analysis is used primarily to map conditional co-occurrence patterns (“which strengths tend to cluster together, and which strengths show the most robust partial associations with self-esteem”), rather than to infer causal influence or mechanism. Although this approach has been preliminarily applied in BPD research, existing network studies have focused almost exclusively on symptom-level interactions (35–37). Few have incorporated positive psychological resources into the same analytical framework, and no study to date has systematically examined how self-esteem and inner strengths interact within a unified system.

To address these gaps, the present study employed network analysis to investigate conditional associations between self-esteem and the ten dimensions of inner strengths among Thai university students with borderline personality features. Based on prior theory and empirical evidence, we expected self-esteem to show a prominent position in this system and to exhibit meaningful connections with key inner strengths; however, we evaluated centrality estimates cautiously given known stability challenges in psychological networks.

**Figure 1:**
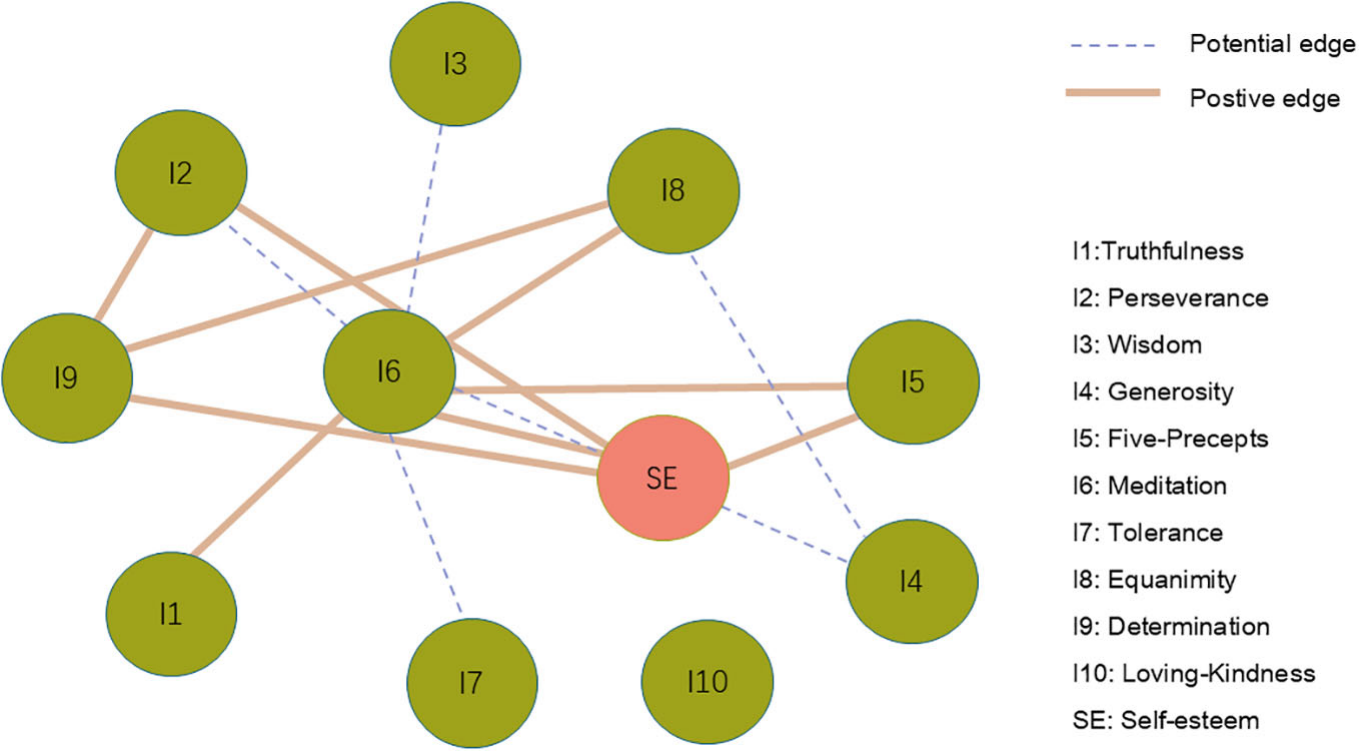
Conceptual model illustrates the hypothesized relationships between variables.

The overall research framework and hypothesized relationships are illustrated in **Figure 1**.

Hypotheses. Given prior theory and empirical work, we hypothesized that (H1) partial associations among inner strengths would be predominantly positive, reflecting a coherent resource system; (H2) self-esteem would show robust positive partial associations with a subset of strengths conceptually linked to self-worth regulation, particularly Determination and Equanimity, and (H3) a prosocial cluster (e.g., Generosity and Loving-kindness) would show a strong positive association. We additionally computed centrality indices as descriptive network characteristics; however, we anticipated that centrality interpretations might be limited by stability constraints and therefore treated them as exploratory.

## 2 Materials and methods

### 2.1 Study design and participants

The present study employed a cross-sectional design and applied network analysis to conduct a secondary analysis of an existing dataset. The original study, titled *“Association Between Pets and Mental Health in University Students with Borderline Personality Disorder Symptoms”* (38), was conducted at Chiang Mai University, Thailand. Participants were recruited through both online and offline advertisements distributed across university campuses and psychiatric outpatient clinics between November 2021 and August 2022.

The inclusion criteria were as follows (1): Thai university students aged 20–30 years; and (2) a score of ≥ 7 on the SI-Bord, indicating a clinically significant level of borderline personality disorder symptoms. Exclusion criteria included (1): comorbid severe psychiatric disorders (e.g., schizophrenia, bipolar disorder) (2); neurological conditions that could affect mental status assessment (e.g., epilepsy, traumatic brain injury); and (3) cognitive impairment that would preclude independent completion of the questionnaires.

Of the 1,244 students initially approached, 693 were excluded (7 declined to provide informed consent; 686 scored ≤ 7 on the SI-Bord). Among the 551 students who met the preliminary eligibility criteria, 205 were further excluded (26 duplicate responses; 179 declined participation), resulting in a final sample of 346 participants included in the original dataset.

For the present study, data from these 346 Thai university students with BPD symptoms were extracted, including measures of the ten dimensions of inner strengths and self-esteem, and subjected to network analysis. Ethical approval for this study was obtained from the Research Ethics Committee of the Faculty of Medicine, Chiang Mai University (Approval No. PSY-2568-0643). This approval pertains to the current secondary analysis of an existing dataset; the original data collection was approved under a separate protocol for the parent study (Approval No. PSY-2566-0502; date of approval: 12 March 2024).

### 2.2 Measurements

#### 2.2.1 Screening instrument for borderline personality disorder

Borderline personality disorder (BPD) symptoms were assessed using the Screening Instrument for Borderline Personality Disorder (SI-Bord) developed by Lohanan et al. (2020). The SI-Bord is a brief self-report measure based on the core diagnostic criteria for BPD outlined in the Diagnostic and Statistical Manual of Mental Disorders, Fifth Edition (DSM-5), and is specifically designed for screening in university student populations (10).

The instrument consists of five items, each corresponding to a core dimension of BPD (1): fear of abandonment (2); unstable interpersonal relationships (3); identity disturbance (4); self-injurious or suicidal behaviors; and (5) affective instability. Responses are rated on a 4-point Likert scale ranging from 0 (“never”) to 3 (“very often”), yielding a total score ranging from 0 to 15, with higher scores indicating greater severity of BPD symptoms.

When a cut-off score of >7 is applied, the SI-Bord demonstrates a sensitivity of 75.00% (95% CI: 47.6–92.7) and a specificity of 73.08% (95% CI: 59.0–84.4) (10). The instrument has shown satisfactory psychometric properties. Using the Structured Clinical Interview for DSM-IV Axis II Disorders (SCID-II) as the gold standard, receiver operating characteristic (ROC) analysis yielded an area under the curve (AUC) of 0.83 (95% CI: 0.715–0.907), indicating moderate to good discriminative validity for BPD (10).

The internal consistency of the SI-Bord is acceptable, with a Cronbach’s α of 0.76 reported in prior research (10). In the present sample, the Cronbach’s α coefficient was also 0.76.

#### 2.2.2 Inner strength-based inventory

Inner strengths were assessed using the Inner Strength-Based Inventory (iSBI). This instrument is designed to evaluate individuals’ levels across ten positive psychological qualities: Truthfulness, Perseverance, Wisdom, Generosity, adherence to the Five Precepts, Meditation, Tolerance, Equanimity, Determination, and Loving-kindness (23). Each dimension is represented by a single item, yielding a total of 10 items. Responses are rated on a 5-point Likert scale, with each item featuring five hierarchically ordered response options reflecting increasing levels of the construct. For example, the meditation item ranges from: “I rarely meditate, or I have never properly meditated before,” to “I try to meditate on some occasions,” “I often meditate but not every day,” “I meditate every day, at a certain time,” and “I meditate every day, at a certain time including some additional available time.”

Total scores range from 10 to 50, with higher scores indicating greater levels of inner strengths. The original study employed the Rasch measurement model to conduct a rigorous psychometric evaluation of the scale (23). The results supported the unidimensionality of the instrument, with all ten items fitting the unidimensional model. Principal component analysis indicated that the primary dimension explained 58.0% of the variance, while the eigenvalue of the first residual contrast was 1.62, below the critical threshold of 2.0, further supporting unidimensionality.

Item fit statistics showed that Infit and Outfit mean square values ranged from 0.68 to 1.68, all within acceptable limits. The iSBI demonstrated good internal consistency, with a Cronbach’s α of 0.86, and an item reliability coefficient of 0.99. Furthermore, no significant differential item functioning (DIF) was observed across age or gender groups, indicating measurement invariance across demographic subgroups (23). Because each Ten Pāramī dimension in the iSBI is assessed with a single item, we address implications for measurement precision and network estimation in the Limitations section.

#### 2.2.3 Rosenberg self-esteem scale

Self-esteem was assessed using the Thai revised version of the Rosenberg Self-Esteem Scale (RSES). The scale consists of 10 items rated on a 4-point Likert scale (1 = strongly disagree to 4 = strongly agree), with total scores ranging from 10 to 40; higher scores indicate higher levels of self-esteem.

The Thai revision primarily addressed a problematic reverse-worded item in the original scale (Item 5: “I wish I could have more respect for myself”) by rephrasing it into a positively worded statement: “I think I am able to give myself more respect.” The revised version comprises six positively worded items and four reverse-worded items.

Confirmatory factor analysis indicated that a unidimensional structure with correlated uniqueness to account for method effects provided a significantly better fit than the original model: χ² = 29.19, df = 19, p = 0.063, GFI = 0.970, TLI = 0.969, NFI = 0.964, CFI = 0.987, SRMR = 0.040, and RMSEA = 0.054 (90% CI: 0.000–0.090), with all indices indicating good to excellent model fit (39).

The scale demonstrated good internal consistency, with a Cronbach’s α of 0.84 in prior research (39). In the present sample, the Cronbach’s α coefficient was 0.86.

### 2.3 Statistical analysis plan

Data preprocessing and descriptive statistical analyses were conducted using SPSS(v.27.0) (40, 41). For demographic variables (age, gender, and academic year), means, standard deviations, frequencies, and percentages were calculated. For the core study variables, including the ten dimensions of inner strengths and self-esteem, means, standard deviations, skewness, and kurtosis were computed to assess distributional characteristics.

Prior to network analysis, bivariate correlation analyses were performed using R (version 4.3.1) (32). Pearson’s product–moment correlation coefficients were calculated to examine the associations between demographic variables (age, gender, academic year) and the ten dimensions of inner strengths as well as self-esteem. Correlation coefficients and their corresponding significance levels (p-values) were reported, with statistical significance indicated using asterisks (e.g., *p* <.05, *p* <.01).

Based on the correlation analyses, a regularized partial correlation network was subsequently estimated and visualized using the qgraph package (version 1.9.8) (42). Network estimation was conducted using the EBICglasso (Extended Bayesian Information Criterion graphical LASSO) method, which applies graphical least absolute shrinkage and selection operator (graphical LASSO) regularization to the partial correlation matrix. This approach shrinks weak and potentially spurious edges to zero, resulting in a sparse and more stable network structure while effectively reducing false-positive edges (43). Such regularization minimizes the risk of overfitting and highlights the most robust and meaningful associations among variables (32).

The network structure was visualized using the *qgraph* package with a spring layout based on the Fruchterman–Reingold algorithm, positioning more strongly related nodes closer together in space (42). In the final undirected network, each node represents a psychological variable, and edges represent partial correlation coefficients, reflecting the unique association between two nodes after controlling for all other variables. Green edges indicate positive associations, whereas red edges indicate negative associations, with edge thickness proportional to the absolute magnitude of the correlation. To characterize overall network properties, we report the number of non-zero edges (i.e., edges retained after regularization), network density, and the mean weights of positive and negative edges.

To quantify the importance of each node within the network, four centrality indices: strength, closeness, betweenness, and expected influence were computed using the *qgraph* (version 1.9.8) and *networktools* (version 1.6.0) packages (44). All centrality metrics were standardized (z-scores) and visualized using centrality plots to describe relative node connectivity. Given known stability challenges, particularly for betweenness and closeness in sparse regularized networks, centrality indices were interpreted cautiously and treated as exploratory when stability criteria were not met.

The accuracy of edge weight estimates was evaluated using nonparametric bootstrap procedures with 500 resamples, generating 95% confidence intervals for each edge. Narrow confidence intervals that do not include zero indicate good precision and stability of the estimated edge weights (45). Edges whose bootstrap CIs excluded zero were treated as the most robust associations for substantive interpretation.

In addition, the stability of centrality indices was assessed using a case-dropping bootstrap approach. By progressively removing subsets of the sample, the variability of centrality estimates across different subsets was examined, and the correlation stability coefficient (CS-coefficient) was computed. According to established guidelines, a CS-coefficient above 0.25 indicates acceptable stability, whereas values above 0.50 indicate good stability (45). Centrality indices with CS-coefficients below 0.25 should be interpreted with caution. There were no missing values on variables included in the network analyses; therefore, all analyses used complete data (n = 346).

## 3 Results

### 3.1 Descriptive statistics and bivariate correlations

**Table 1** indicates that a total of 346 Thai university students with BPD symptoms were included in the present study. The mean age of the participants was 21.6 years (SD = 2.23). The majority of participants were female (88%) and in their third year of study (27.5%).

**Table 1:** Sample characteristics (n = 346).

Characteristic	Category	Value
Age, M ± SD	–	21.60 ± 2.24
Gender, n (%)	Male	88 (25.4%)
	Female	258 (74.6%)
Year of Study, n (%)	1st year	33 (9.5%)
	2nd year	85 (24.6%)
	3rd year	95 (27.5%)
	4th year	75 (21.7%)
	Above 4th year	58 (16.8%)

n, number of participants; M, mean; SD, standard deviation.

Descriptive statistics for all study variables are presented in **Table 2**. Across the ten dimensions of inner strengths, mean scores ranged from 1.49 (Meditation) to 3.48 (Generosity), with standard deviations ranging from 0.78 (Meditation) to 1.27 (Truthfulness). Skewness and kurtosis analyses indicated that most variables approximated a symmetric distribution. However, the Meditation dimension exhibited a pronounced positive skew (skewness = 1.98) and high kurtosis (kurtosis = 4.58). The mean self-esteem score was 25.84 (SD = 5.15), with an approximately normal distribution.

**Table 2:** Descriptive statistics (n = 346).

Variables	Mean (SD)	Skewness	Kurtosis
Truthfulness	3.20 ± 1.27	0.18	−1.37
Perseverance	2.41 ± 1.03	0.61	−0.05
Wisdom	2.95 ± 1.19	−0.11	−0.80
Generosity	3.48 ± 1.27	−0.49	−1.08
Five Precepts	2.82 ± 1.22	0.03	−0.91
Meditation	1.49 ± 0.78	1.98	4.58
Tolerance	3.17 ± 1.13	−0.07	−1.05
Equanimity	2.95 ± 1.01	0.03	−0.64
Determination	3.02 ± 1.11	0.18	−0.96
Loving-Kindness	3.23 ± 1.23	−0.45	−1.03
Self-esteem	25.84 ± 5.15	−0.02	1.15

n, number of participants; M, Mean; SD, Standard Deviation.

As shown in **Table 3**, bivariate correlation analyses revealed that age was positively associated with Loving-kindness (r = 0.27, p <.001) and negatively associated with self-esteem (r = −0.11, p <.05). Females scored significantly higher than males on Generosity (r = 0.20, p <.01) and Loving-kindness (r = 0.27, p <.001), whereas males reported significantly higher self-esteem than females (r = −0.12, p <.05).

**Table 3:** Correlation matrix of demographic characteristics and variables.

Variables	1	2	3	4	5	6	7	8	9	10	11	12	13	14
1. Sex	–													
2. Age	0.00	–												
3. Year study	0.10	0.65 ***	–											
4. Tru	0.08	0.07	−0.07	–										
5. Per	−0.01	−0.00	−0.03	0.19 ***	–									
6. Wis	0.11	0.01	0.03	0.07 **	0.32 ***	–								
7. Gen	0.20 ***	−0.06	0.06	0.19 ***	0.19 ***	0.27 ***	–							
8. Pre	0.13 *	−0.01	−0.05	0.09	0.22 ***	0.23 ***	0.21 ***	–						
9. Med	−0.03	−0.07	−0.05	0.08	0.21 ***	0.15 **	0.07	0.25 ***	–					
10. Tol	0.08	0.06	0.10	0.18 ***	0.18 ***	0.24 ***	0.28 ***	0.10	0.06	–				
11. Equ	−0.10	0.02	0.09	−0.07	−0.02	0.15 **	0.13 **	0.20 ***	0.00	0.22 ***	–			
12. Det	−0.00	−0.03	0.08	0.09	0.23 ***	0.30 ***	0.21 ***	0.24 ***	0.25 ***	0.17 **	0.18 ***	–		
13. Lov	0.27 ***	−0.03	0.02	0.18 ***	0.20 ***	0.12 *	0.40 ***	0.03 ***	0.04	0.14 **	0.04	0.27 ***	–	
14. Se	-0.12*	-0.11*	-0.05	0	0.26***	0.27***	0.08	0.21***	0.27***	0.14*	0.32***	0.4***	0.13*	

*p < 0.05; **p < 0.01; ***p < 0.001.

The ten inner strengths were generally positively intercorrelated, suggesting a degree of structural coherence among these psychological qualities. In addition, self-esteem was significantly and positively correlated with multiple dimensions of inner strengths, with relatively stronger associations observed for Equanimity (r = 0.32, p <.001), Wisdom (r = 0.27, p <.001), and Meditation (r = 0.27, p <.001).

### 3.2 Network structure

**Figure 2:**
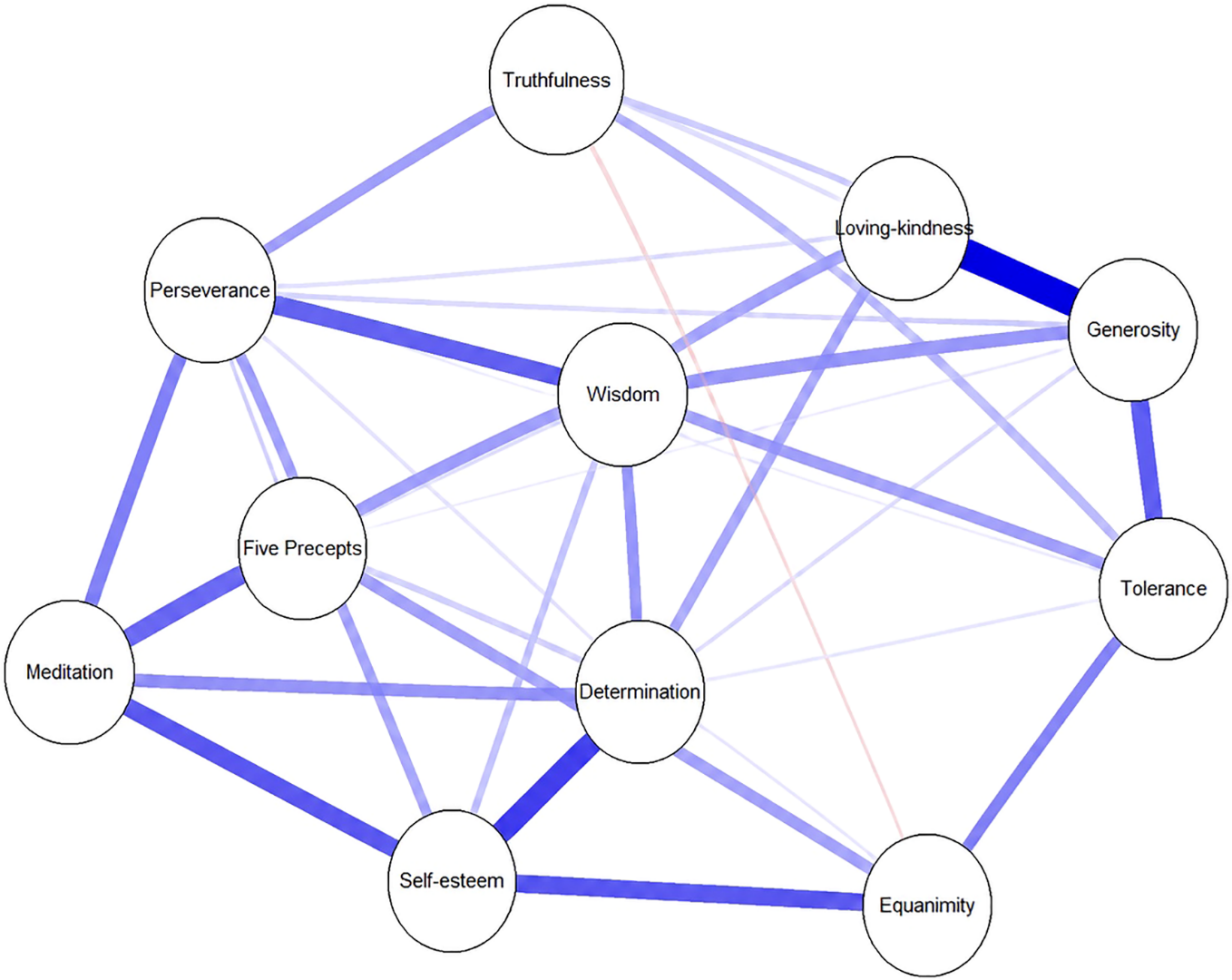
Network of Self-esteem and Inner Strength in BPD. Each circle (node) represents variables, Lines (edges) indicate relationships between nodes. These relationships are represented by weight values in the network that are based on partial correlations. Thicker and darker edges signify stronger relationships. Green edges denote positive relationships, and red edges represent negative relationships. Negative edges (red) are shown for completeness but are considered exploratory given limited bootstrap precision.

**Figure 2** presents the regularized partial correlation network consisting of eleven nodes—ten inner strengths and self-esteem. Among the 55 possible edges in the 11-node network, 34 were non-zero (density = 0.618), including 33 positive and 1 negative edge. The mean weight of positive edges was 0.108, whereas the mean weight of negative edges was −0.046.

The strongest association in the network was observed between Generosity and Loving-kindness (r = 0.33). This was followed by the associations between self-esteem and Equanimity (r = 0.28), and between self-esteem and Determination (r = 0.26).

Within the domain of inner strengths, weak but stable negative associations were identified between Perseverance and Equanimity (r = −0.15), as well as between Truthfulness and Equanimity (r = −0.10). The full weighted partial correlation matrix is presented in **Supplementary Table 1**.

### 3.3 Centrality analysis

**Figure 3:**
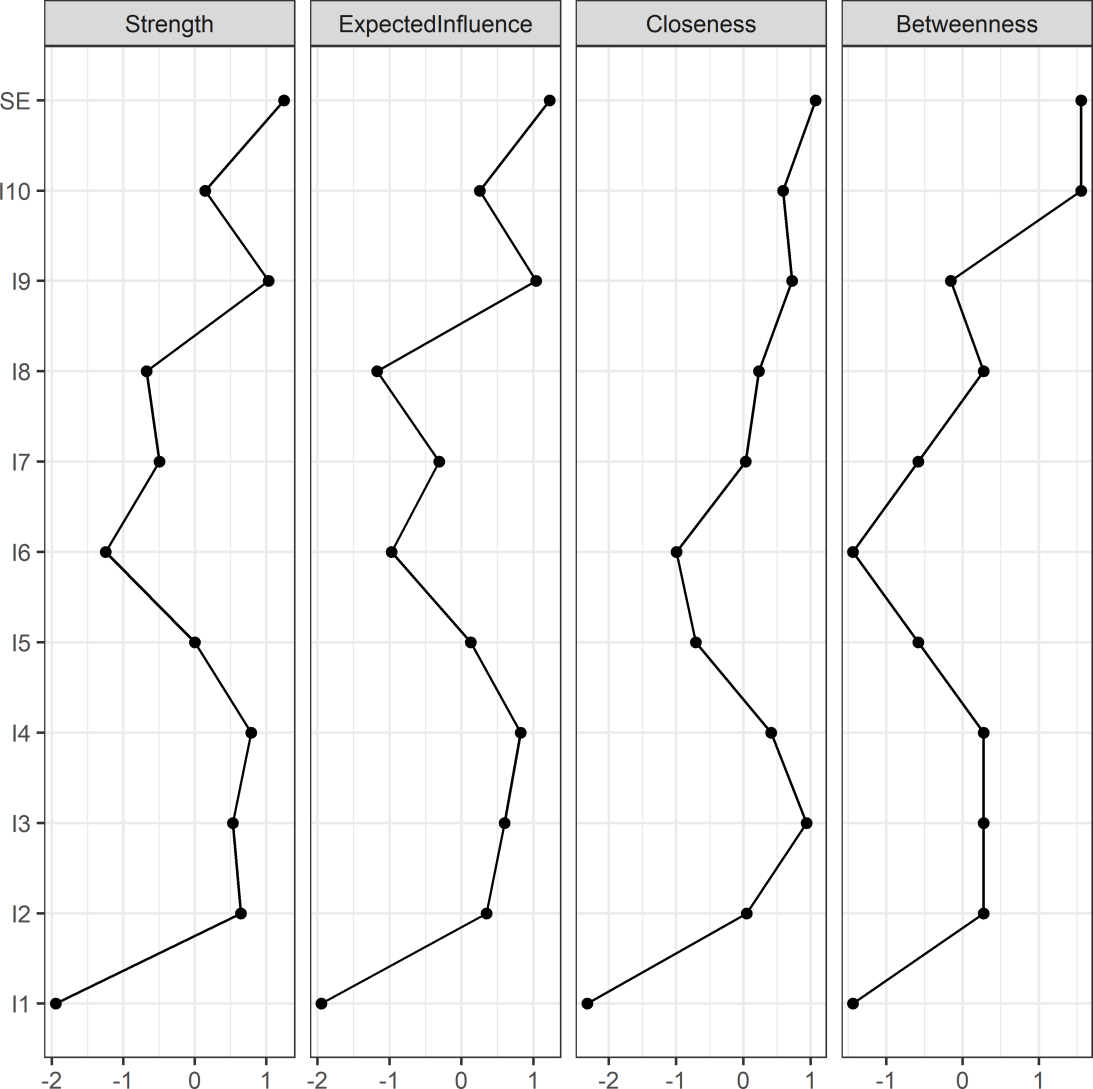
Centrality indices. All centrality values are z standardized. I1 = Truthfulness; I2 = Perseverance; I3 = Wisdom; I4 = Generosity; I5 = Five Precepts; I6 = Meditation; I7 = Tolerance; I8 = Equanimity; I9 = Determination; I10 = Loving-Kindness; SE = Self-esteem.

The results of the centrality analysis are presented in **Figure 3**. Consistent with the study hypothesis, self-esteem exhibited the highest centrality across all indices, indicating its role as the core driver of the network.

In terms of strength centrality, self-esteem (z = 1.24) showed the highest value, followed by Determination (z = 1.03), suggesting that these variables have the strongest direct connections with other nodes in the network.

Furthermore, both self-esteem (z = 1.56) and Loving-kindness (z = 1.56) demonstrated the highest betweenness centrality, highlighting their critical roles as connectors or bridges linking different domains within the network.

Regarding expected influence, self-esteem (z = 1.22) and Determination (z = 1.03) exhibited relatively high positive values, indicating that increases in these traits may exert broad positive effects across the network. In contrast, Truthfulness (z = −1.95) and Equanimity (z = −1.17) showed relatively high negative expected influence, suggesting that these nodes may exert substantial inhibitory effects on network activation when negative associations are considered.

With respect to closeness centrality, self-esteem (z = 1.07) was notably higher than that of other nodes, indicating that it may substantially reduce the path lengths between variables and facilitate more efficient transmission of influence throughout the network.

Overall, these findings suggest that the network structure is relatively centralized, with clearly identifiable hub nodes. However, given the limited stability of strength centrality and expected influence in subsequent stability analyses, these results should be interpreted with caution. Detailed results are provided in **Supplementary Table 2**.

### 3.4 Gender specific analyses

Given the predominance of female participants, exploratory gender-stratified analyses were conducted. The strongest edges in the female subsample were similar to those observed in the overall network (**Supplementary Table 3**), although fewer edges were retained and the precision of some edge-weight estimates likely decreased due to the reduced sample size. In the female subsample, Self-esteem and Perseverance showed the highest centrality estimates across indices (**Supplementary Table 4**), with Determination also ranking relatively high in closeness; however, because centrality stability was not assessed separately by gender, these rankings should be interpreted as descriptive and hypothesis-generating. In contrast, the male network could not be reliably estimated because regularization yielded a sparse/near-empty structure.

In contrast, the male subsample (n = 88) was limited by small sample size. Following regularization, most edge weights were shrunk to zero, resulting in uninformative centrality indices (all values = 0.000; **Supplementary Table 4**) and precluding estimation of a stable male-specific network structure. Therefore, no firm conclusions regarding gender differences can be drawn from these exploratory analyses (see **Supplementary Table 4**).

### 3.5 Accuracy and stability of the network

**Figure 4:**
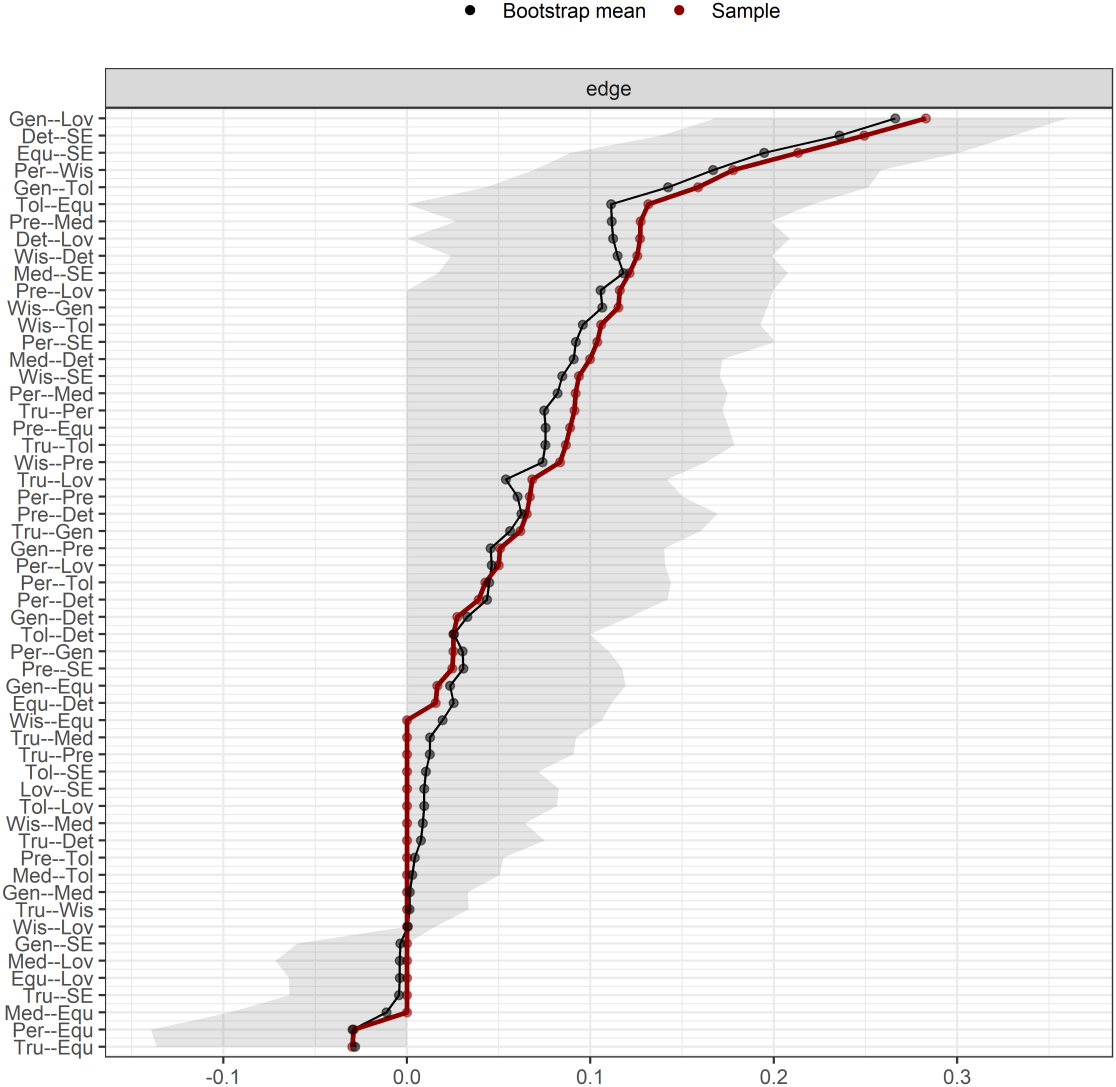
Accuracy of the edge-weight estimates. Black dots represent the bootstrap mean of edge weights, and red dots indicate the original sample estimates. Gray areas denote the 95% bootstrap confidence intervals (CIs) for each edge. Several stronger edges show relatively narrower CIs, whereas many smaller edges have CIs that include or touch zero, indicating greater uncertainty in the magnitude (and in some cases the direction) of those edge-weight estimates.

**Figure 4** displays the bootstrap 95% confidence intervals (CIs) for the estimated edge weights. The bootstrap results indicated that several key edges were robustly different from zero, whereas many smaller edges had CIs that included or touched zero, suggesting greater uncertainty in their magnitude and, for some edges, in their direction. The strongest robust association was observed between Generosity and Loving-kindness (mean = 0.265, 95% CI [0.165, 0.359]), followed by Determination and Self-esteem (mean = 0.234, 95% CI [0.134, 0.334]) and Equanimity and Self-esteem (mean = 0.196, 95% CI [0.099, 0.286]). Additional edges with CIs excluding zero included Perseverance–Wisdom, Generosity–Tolerance, Determination–Lovingkindness, Wisdom–Determination, Meditation–Self-esteem, and Meditation–Determination. Overall, bootstrap CIs support higher confidence in the strongest edges, while weaker connections should be interpreted cautiously.

In addition, the CIs for the strongest edges were relatively narrow, indicating good precision and stability in the estimation of associations between key nodes. This further enhances confidence in the interpretability of the identified network structure. However, some edges exhibited relatively wide confidence intervals, suggesting a degree of uncertainty in the magnitude of those edge weight estimates.

**Figure 5:**
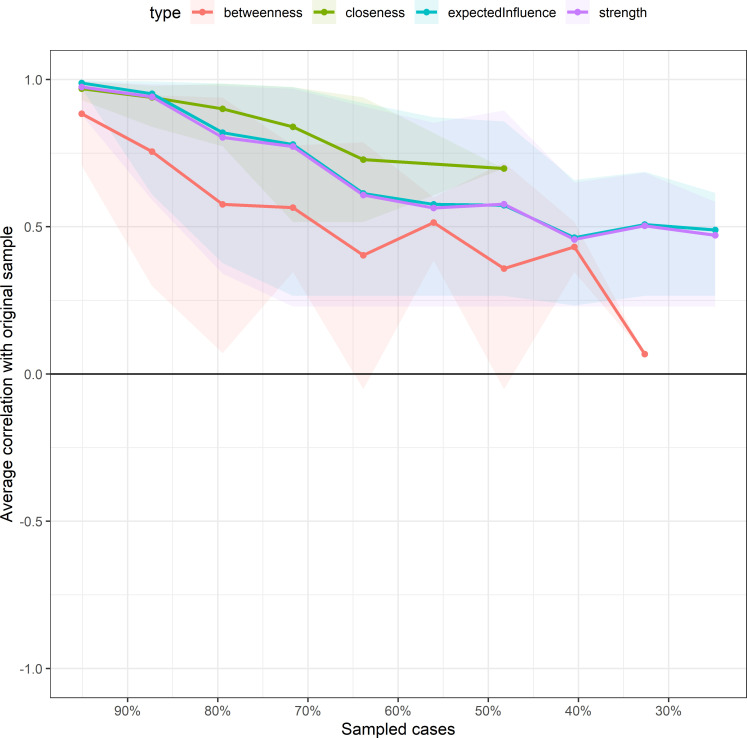
Stability of centrality indices (case-dropping bootstrap). The x-axis shows the proportion of cases remaining in each subsample, and the y-axis shows the correlation between centrality estimates from the full sample and those from the case-dropped subsamples. Lines correspond to centrality measures (strength, expected influence, and closeness); shaded bands indicate variability across bootstrap replications.

**Figure 5** presents the case-dropping bootstrap results for centrality stability. The correlation stability coefficient, CS(cor = 0.7), was 0.13 for both strength and expected influence, which is below the recommended minimum of 0.25 (and well below the preferred value of 0.50). This indicates that the rank-ordering of nodes on these centrality indices is not sufficiently stable and should be interpreted with caution.

Closeness and betweenness centrality showed very low stability (CS values close to zero), suggesting that these indices are not reliable for interpretation in the present network. Consistent with prior recommendations in psychological network analysis, closeness and betweenness often exhibit poor stability, and thus substantive conclusions should not be based on these indices.

## 4 Discussion

The present study applied a network analytic approach to examine the partial association structure among ten inner strengths and self-esteem in Thai university students screening positive for BPD symptoms. The estimated network was dominated by positive connections, and several theoretically meaningful associations emerged. Although self-esteem was connected to multiple strengths in the estimated network, interpretations that rely on node importance (e.g., identifying “most central” nodes) should be made cautiously given the limited stability of centrality estimates (see Robustness). Importantly, the estimated edges represent cross-sectional partial associations (conditional co-occurrence) and do not support directional or causal inference.

From a structural perspective, the predominance of positive edges suggests that inner strengths may cluster in a broadly reinforcing manner. This pattern is consistent with positive-psychology perspectives on synergistic psychological resources and with the Theravāda Buddhist framing of the Ten Pāramīs as mutually supportive qualities (46–49). At the same time, bootstrap edge-weight accuracy indicated that only a subset of edges was estimated with relatively high precision (i.e., bootstrap CIs excluding zero), whereas many smaller edges had CIs that included or touched zero. Accordingly, substantive interpretation should focus primarily on the strongest and most consistently estimated connections, while treating weaker associations as provisional.

At the level of specific associations, the most robust edge connected Generosity and Loving-kindness, suggesting a coherent prosocial cluster. This finding aligns with Buddhist psychological accounts in which generosity can be understood as a behavioral expression of benevolent intention (49–52), while loving-kindness may motivate and sustain prosocial action (53). In students screening positive for BPD symptoms, this linkage highlights a potentially important resource domain that can be conceptualized and cultivated within a strengths-based framework rather than exclusively from a deficit perspective. In the Thai Theravāda context, dana (generosity) and metta (loving-kindness) are highly salient, socially reinforced practices; their strong co-occurrence may therefore reflect both shared motivational foundations and culturally scaffolded behavioral opportunities for cultivating prosociality.

Beyond prosocial strengths, self-esteem showed its most precisely estimated links with Determination and Equanimity (i.e., edges with bootstrap CIs excluding zero). This pattern suggests that self-respect/self-worth in this population may be most consistently related to the capacity to sustain wholesome intentions and follow through (adhiṭṭhāna) and the capacity to remain balanced and nonreactive amid fluctuating mind-states (upekkhā) (54). Conceptually, these associations map onto skill domains emphasized in DBT and mindfulness-based cognitive-behavioral approaches (MCBT) including committed values-consistent action and nonreactive awareness while remaining compatible with Buddhist-virtue interpretations of these qualities (55, 56) (57). Culturally, these strengths may be especially relevant for Thai students navigating interpersonal sensitivity and affective volatility, because determination can support continuity of values-consistent action, whereas equanimity may support nonreactive balance under emotionally triggering contexts. Nevertheless, the present results reflect cross-sectional partial associations and should not be interpreted as evidence that modifying one node will necessarily produce downstream change in the broader network.

In contrast, interpretations regarding functional tensions among strengths require caution in the present dataset. Although some edges involving Truthfulness, Perseverance, and Equanimity were estimated as negative, their bootstrap CIs included or touched zero, indicating limited precision and uncertainty regarding whether these negative associations are reliable. Accordingly, these negative edges are treated as preliminary and are not emphasized as substantive findings, and it remains unclear whether the apparent trade-offs between effortful striving and acceptance reflect stable dynamics in students with BPD symptoms or instead are context-dependent, measurement-related, or sample-specific. Future work using larger samples and longitudinal designs is needed to clarify whether and under what conditions certain virtues may conflict in practice, and whether psychological flexibility moderates these relations.

Cultural and measurement considerations are also important. In the Thai Buddhist context, certain virtues (e.g., truthfulness and commitment-keeping) may be especially salient and therefore more susceptible to social desirability or internalized moral expectations (58). These factors could influence both mean levels and observed associations among strengths. Future studies should examine measurement invariance, potential construct redundancy among inner-strength dimensions, and whether the observed network structure replicates across samples with different demographic compositions and levels of BPD symptom severity.

Finally, given the predominance of female participants, exploratory gender stratified analyses were conducted to examine whether the overall pattern of associations was broadly similar by gender. The network estimated in the female subsample was broadly consistent with the full-sample structure, with the strongest edges showing similar patterns, although fewer edges were retained and precision likely decreased due to the reduced sample size. Because centrality stability was not evaluated separately by gender and centrality indices showed limited stability in the full sample, we do not emphasize centrality-based subgroup rankings and treat any such patterns as descriptive and hypothesis-generating only. In the male subsample (n = 88), regularization yielded a sparse/near-empty network, precluding reliable estimation of a male-specific structure and preventing meaningful inference regarding gender differences. Accordingly, gender-stratified findings are reported descriptively and should be interpreted as exploratory.

### 4.1 Robustness and clinical interpretation

Bootstrap results suggested comparatively better precision for the strongest edges, whereas many weaker edges showed substantial uncertainty (CIs including/touching zero). The case-dropping bootstrap indicated limited stability of centrality estimates (CS (cor = 0.7) = 0.13 for strength and expected influence). Although we initially expected self-esteem to emerge as the central node, the low CS coefficient indicates that any such ranking cannot be interpreted reliably in the present sample. Therefore, centrality-based conclusions should be considered exploratory and hypothesis generating. Therefore, substantive interpretation in the revised manuscript is anchored primarily to robust edges with bootstrap CIs excluding zero, rather than to centrality rankings. Although global metrics such as closeness and betweenness are intuitively appealing as indicators of system-wide reach or bottleneck roles (33, 59), they are particularly prone to instability in sparse, regularized psychological networks. Thus, clinical interpretation should place greater weight on the most robust and consistently estimated edges and test these candidate associations in larger samples and in longitudinal or interventional designs.

### 4.2 Implications

Given the above robustness results, intervention implications should prioritize the most precisely estimated associations, particularly Generosity and Loving-kindness, and the links between Self-esteem and both Determination and Equanimity. In practice, supportive programs for students screening positive for BPD symptoms could integrate structured kindness giving exercises paired with Loving kindness reflection, alongside brief determination based commitment practices and equanimity-based mindfulness exercises for returning to balance under stress (55, 60). These components map conceptually onto skill domains emphasized in DBT and mindfulness-based cognitive-behavioral approaches (MCBT) and could be delivered via an 8 week group, workshops, or online modules with peer-mentoring check-ins, while monitoring change over time (61–66). As an example, an 8-week strengths-based group program could be structured around (i) psychoeducation on self-worth instability and culturally grounded strengths, (ii) weekly behavioral practice of generosity (planned giving/helping) combined with guided loving-kindness exercises, (iii) values/goal clarification with determination-based commitment plans and between-session check-ins, and (iv) equanimity-oriented mindfulness practices focused on nonreactivity during interpersonal triggers, with brief homework logs to support skill generalization (61–66). However, given the uncertainty of many weaker edges and the instability of centrality indices, these targets should be treated as candidate, culturally congruent directions for strengths-based support rather than definitive intervention mechanisms. Future longitudinal and intervention studies are needed to evaluate whether strengthening these candidate resources is followed by improvements in self-esteem and broader changes in the inner-strength network.

### 4.3 Limitations and future directions

Several limitations should be noted. First, the cross-sectional design precludes inferences about temporal ordering or causal relations among inner strengths and self-esteem. Second, the limited stability of centrality estimates indicates that node-ranking interpretations require replication, including stability assessment in subgroup analyses before drawing gender-specific conclusions. Third, many smaller edges were imprecisely estimated, suggesting that fine-grained interpretations of weaker connections should be treated as provisional. Fourth, gender imbalance limited interpretability of male-specific networks, highlighting the need for larger and more demographically balanced samples. Future studies should prioritize larger samples, assess potential construct redundancy and measurement invariance, and use longitudinal or experimental approaches to evaluate whether strengthening specific virtues (e.g., generosity/loving-kindness or determination/equanimity) is followed by broader changes in self-esteem and network structure. Fifth, each inner-strength dimension was assessed using a single iSBI item, which may limit reliability, constrain discriminant validity among strengths, and introduce measurement error that can attenuate or destabilize edge estimates.

Sixth, participants were recruited via convenience sampling from a single university setting, which may limit generalizability to other Thai student populations or clinical BPD samples.

Seventh, potential uncontrolled confounders (e.g., depression, anxiety, trauma history, or other contextual stressors) were not modeled and may influence observed partial associations; future studies should incorporate broader clinical/contextual variables and test whether the identified robust edges replicate after such adjustment.

## 5 Conclusion

Using a network analytic approach, the present study examined the structural relationships between ten dimensions of inner strengths and self-esteem among Thai university students with symptoms of Borderline Personality Disorder. The findings indicated that robust associations with Determination and Equanimity, alongside a prosocial cluster linking Generosity and Loving-kindness, highlight a small set of potentially meaningful psychological resources. These findings reflect cross-sectional conditional co-occurrence patterns and should not be interpreted as directional, predictive, or causal pathways. However, given the cross-sectional design and the limited stability of centrality estimates, these findings should be interpreted as exploratory and hypothesis generating rather than causal. Overall, the results provide a culturally informed basis for strengths-based approaches in university mental health settings, while underscoring the need for longitudinal and intervention research to test whether strengthening these resources leads to sustained improvements in self-esteem and related outcomes.

## Data Availability

The raw data supporting the conclusions of this article will be made available by the authors, without undue reservation.
