# Supplementary material for: Self-esteem and inner strengths: a network study in Thai university students with borderline personality disorder symptoms
Source: Front Psychiatry. 2026 Jul 7;17:1847294. doi: 10.3389/fpsyt.2026.1847294 (PMC13385106; doi:10.3389/fpsyt.2026.1847294)
Supplement: Supplementary file 1 [file Table1.docx]

**Table S1.** Correlation Matrix of Inner Strength Dimensions and Self-esteem.

|  | **I1** | **I2** | **I3** | **I4** | **I5** | **I6** | **I7** | **I8** | **I9** | **I10** | SE |
| --- | --- | --- | --- | --- | --- | --- | --- | --- | --- | --- | --- |
| I1 | - |  |  |  |  |  |  |  |  |  |  |
| I2 | 0.12 | - |  |  |  |  |  |  |  |  |  |
| I3 | -0.03 | 0.20 | - |  |  |  |  |  |  |  |  |
| I4 | 0.09 | 0.04 | 0.16 | - |  |  |  |  |  |  |  |
| I5 | 0.03 | 0.09 | 0.11 | 0.05 | - |  |  |  |  |  |  |
| I6 | 0.04 | 0.10 | -0.01 | 0.02 | 0.19 | - |  |  |  |  |  |
| I7 | 0.14 | 0.08 | 0.12 | 0.17 | -0.04 | 0.00 | - |  |  |  |  |
| I8 | -0.10 | -0.15 | 0.02 | 0.09 | 0.16 | -0.11 | 0.19 | - |  |  |  |
| I9 | 0.02 | 0.03 | 0.14 | 0.04 | 0.05 | 0.14 | 0.03 | 0.05 | - |  |  |
| I10 | 0.09 | 0.07 | -0.07 | 0.33 | 0.17 | -0.10 | 0.01 | -0.07 | 0.17 | - |  |
| SE | -0.05 | 0.16 | 0.11 | -0.10 | 0.01 | 0.17 | 0.00 | 0.28 | 0.26 | 0.05 | - |
| Note. Values are Pearson’s correlation coefficients. I1 = Truthfulness; I2 = Perseverance; I3 = Wisdom; I4 = Generosity; I5 = Five-Precepts; I6 = Meditation; I7 = Tolerance; I8 = Equanimity; I9 = Determination; I10 = Loving-kindness; SE = Self-esteem. | | | | | | | | | | | |

| Table S2. Centrality Analysis of Variables | | | | |
| --- | --- | --- | --- | --- |
| **Table S2.** Centrality Analysis of Variables | | | | |
| Variable | Betweenness | Closeness | Strength | Expected influence |
| **I1** | -.144 | -2.32 | -1.95 | -1.95 |
| **I2** | 0.27 | 0.05 | 0.64 | 0.35 |
| **I3** | 0.27 | 0.93 | 0.53 | 0.60 |
| **I4** | 0.27 | 0.41 | 0.78 | 0.82 |
| **I5** | -0.58 | -0.71 | 0.00 | 0.13 |
| **I6** | -1.44 | -1.00 | -1.25 | -0.97 |
| **I7** | -0.58 | 0.03 | -0.50 | -0.31 |
| **I8** | 0.27 | 0.23 | -0.68 | -1.17 |
| **I9** | -0.16 | 0.72 | 1.03 | 1.03 |
| **I10** | 1.56 | 0.59 | 0.14 | 0.25 |
| **SE** | 1.56 | 1.07 | 1.24 | 1.22 |
| **Note.** I1 = Truthfulness; I2 = Perseverance; I3 = Wisdom; I4 = Generosity; I5 = Five-Precepts; I6 = Meditation; I7 = Tolerance; I8 = Equanimity; I9 = Determination; I10 = Loving-kindness; SE = Self-esteem. Centrality values are z-standardized. Centrality indices should be interpreted cautiously because stability analyses indicated limited stability in the present network (see case-dropping bootstrap results in the main text/Figure 5). | | | | |

**Table S3.** **EBICglasso Regularized Partial Correlation (Weight) Matrix (Full Sample)**

|  | I1 | I2 | I3 | I4 | I5 | I6 | I7 | I8 | I9 | I10 |
| --- | --- | --- | --- | --- | --- | --- | --- | --- | --- | --- |
| **I1** | 0.000 | 0.110 | 0.000 | 0.063 | 0.000 | 0.000 | 0.086 | -0.046 | 0.000 | 0.044 |
| **I2** | 0.110 | 0.000 | 0.189 | 0.062 | 0.080 | 0.137 | 0.032 | 0.000 | 0.045 | 0.039 |
| **I3** | 0.000 | 0.189 | 0.000 | 0.122 | 0.069 | 0.000 | 0.102 | 0.000 | 0.116 | 0.000 |
| **I4** | 0.063 | 0.062 | 0.122 | 0.000 | 0.022 | 0.000 | 0.193 | 0.000 | 0.050 | 0.311 |
| **I5** | 0.000 | 0.080 | 0.069 | 0.022 | 0.000 | 0.172 | 0.000 | 0.104 | 0.068 | 0.107 |
| **I6** | 0.000 | 0.137 | 0.000 | 0.000 | 0.172 | 0.000 | 0.000 | 0.000 | 0.125 | 0.000 |
| **I7** | 0.086 | 0.032 | 0.102 | 0.193 | 0.000 | 0.000 | 0.000 | 0.148 | 0.031 | 0.000 |
| **I8** | -0.046 | 0.000 | 0.000 | 0.000 | 0.104 | 0.000 | 0.148 | 0.000 | 0.027 | 0.000 |
| **I9** | 0.000 | 0.045 | 0.116 | 0.050 | 0.068 | 0.125 | 0.031 | 0.027 | 0.000 | 0.109 |
| **I10** | 0.044 | 0.039 | 0.000 | 0.311 | 0.107 | 0.000 | 0.000 | 0.000 | 0.109 | 0.000 |
| **SE** | 0.000 | 0.112 | 0.074 | 0.000 | 0.000 | 0.189 | 0.000 | 0.194 | 0.232 | 0.000 |
| **Note.** I1 = Truthfulness; I2 = Perseverance; I3 = Wisdom; I4 = Generosity; I5 = Five-Precepts; I6 = Meditation; I7 = Tolerance; I8 = Equanimity; I9 = Determination; I10 = Loving-kindness; SE = Self-esteem. Entries are regularized partial correlation coefficients estimated using EBICglasso. Zero values indicate edges shrunk to zero by regularization (i.e., not retained as non-zero edges). Edge-weight accuracy was evaluated in the main analysis using nonparametric bootstrap 95% confidence intervals (CIs); edges whose CIs excluded zero were prioritized in substantive interpretation. | | | | | | | | | | |

**Table S4.** Centrality Analysis of Gender (Female)

| Variable | Betweenness | Closeness | Strength | Expected influence |
| --- | --- | --- | --- | --- |
| **I1** | -1.311 | -2.028 | -1.699 | -1.699 |
| **I2** | 1.144 | 0.994 | 1.429 | 1.492 |
| **I3** | -1.311 | -0.077 | -0.192 | -0.192 |
| **I4** | 0.530 | -0.033 | 0.083 | 0.083 |
| **I5** | -1.311 | -0.699 | -0.568 | -0.568 |
| **I6** | -0.391 | 0.537 | 0.791 | 0.791 |
| **I7** | -0.084 | -0.938 | -0.673 | -0.673 |
| **I8** | 0.530 | 0.221 | -0.853 | -0.853 |
| **I9** | 0.837 | 1.244 | 0.630 | 0.630 |
| **I10** | -0.084 | -0.150 | -0.462 | -0.462 |
| **SE** | 1.451 | 1.248 | 1.451 | 1.451 |
| **Note.** I1 = Truthfulness; I2 = Perseverance; I3 = Wisdom; I4 = Generosity; I5 = Five-Precepts; I6 = Meditation; I7 = Tolerance; I8 = Equanimity; I9 = Determination; I10 = Loving-kindness; SE = Self-esteem. Centrality values are z-standardized within the female subsample and are reported descriptively. Centrality stability was not assessed separately by gender; therefore, these indices should be interpreted as exploratory/hypothesis-generating only. | | | | |
